# Effect of endoscopic therapy and drug therapy on prognosis and rebleeding in patients with esophagogastric variceal bleeding

**DOI:** 10.1038/s41598-024-57791-8

**Published:** 2024-03-28

**Authors:** Jing-jing Jiang, Chun Gao, Jun-feng Mao, Guo-yuan Yang, Jun Huang, Xiao-hui Yu, Yong Tan, Jiu-cong Zhang, Xiao-feng Zheng

**Affiliations:** 1 Department of Gastroenterology, The 940 Hospital of Joint Logistic Support Forces of PLA, Qilihe District, 333Rd Binhenan Road, Lanzhou, 730050 Gansu China; 2 The First School of Clinical Medical, Gansu University of Chinese Medicine, Lanzhou, 730000 Gansu China; 3 Department of Nuclear Medicine, The 940 Hospital of Joint Logistic Support Forces of PLA, Lanzhou, 730050 Gansu China; 4 Department of Gastroenterology, The Second Hospital of Lanzhou University, Lanzhou, 730030 Gansu China

**Keywords:** Esophagogastric variceal bleeding, Endoscopic therapy, Drug therapy, Propensity score matching, Gastroenterology, Risk factors

## Abstract

Esophagogastric variceal bleeding (EVB) is one of the common digestive system emergencies with poor prognosis and high rate of rebleeding after treatment. To explore the effects of endoscopic therapy and drug therapy on the prognosis and rebleeding of patients with EVB, and then select better treatment methods to effectively improve the prognosis. From January 2013 to December 2022, 965 patients with EVB who were hospitalized in gastroenterology Department of the 940 Hospital of Joint Logistic Support Forces of PLA were retrospectively analyzed. Patients were divided into endoscopic treatment group (ET, n = 586) and drug treatment group (DT, n = 379). Propensity score matching (PSM) analysis was performed in both groups, and the general information, efficacy and length of hospital stay were recorded. The patients were followed up for 3 months after bleeding control to determine whether rebleeding occurred. There were 286 cases in each group after PSM. Compared with DT group, ET had higher treatment success rate (*P* < 0.001), lower rebleeding rate (*P* < 0.001), lower mortality rate within 3 months, and no significant difference in total hospital stay (*P* > 0.05). Compared with drug therapy, endoscopic treatment of EVB has short-term efficacy advantages, and can effectively reduce the incidence of rebleeding and mortality within 3 months.

## Introduction

Esophagogastric variceal bleeding (EVB) is one of the common digestive system emergencies with poor prognosis and a high rate of rebleeding after treatment^1^. Studies have shown that for untreated patients with EVB, the rate of rebleeding within 1–2 years is as high as 60%, and the 6-week fatality rate is as high as 20%^2,3^.Therefore, active and effective treatment is very important for reducing EVB rebleeding and mortality. Clinically, for patients highly suspected of EVB, early application of portal pressure reduction and antibacterial drugs is the primary treatment plan, while gastroscopy is still the main method for definite diagnosis of EVB^4^. So far, systematic reviews and meta-analyses have failed to reach consistent conclusions on the efficacy comparison of the above two treatments^3,5^. Therefore, how to optimize the above treatment methods, still need to carry out related clinical research.

Based on this, this study compared the effects of endoscopic therapy and drug therapy on the prognosis and rebleeding of patients with EVB, hoping to provide certain theoretical basis for clinicians to choose better treatment plans for patients with EVB.

## Methods

### Clinical information

A total of 965 patients with EVB admitted to the Gastroenterology Department of the 940 Hospital of Joint Logistic Support Forces of PLA from January 2013 to December 2022 were selected to collect their detailed clinical data. Baseline information included patient gender, age, smoking history, drinking history, underlying disease, Child–Pugh grade, primary disease, and treatment. Outcome, length of hospital stay, rate of early rebleeding, rate of delayed rebleeding, and mortality within 3 months after treatment were included. Patients were divided into endoscopic treatment group (ET) and drug treatment group (DT) according to the main treatment modalities.

*Inclusion criteria*: (i) 18–80 years; (ii) Meet the relevant diagnostic criteria for EVB: Under endoscopy, active varicose vein bleeding (oozing or spraying), thrombocephalic head, or varicose veins were present without any other lesions that could explain the bleeding.

*Exclusion criteria*: (i) those who do not meet the above diagnostic criteria; (ii) Patients with haemorrhagic diseases of the hematopoietic system or serious diseases of the heart, brain, kidney and other important organs; (iii) Patients with incomplete clinical data or no follow-up within 3 months after treatment; (iv) Patients receiving medication combined with endoscopic therapy.

This study was approved by the 940 Hospital of Joint Logistic Support Forces of PLA (approval number: 2023KYLL160) before its formal implementation. All participants in the study and/or their legal guardians were informed and consented to our study. The research programme complies with the ethical guidelines of the Declaration of Helsinki.

## Treatment methods

### ET group

Endoscopic treatment included endoscopic variceal ligation (EVL), endoscopic injection sclerotherapy (EIS) and endoscopic tissue adhesive injection.

#### EVL

Mainly used for GOV1 EVB patients, six or seven ring ligation device is commonly used, and the first ligation interval of 2 to 4 weeks can be repeated ligation or sclerosing agent injection and other sequential treatment until the varicose veins disappear or basically disappear.

#### EIS

Mainly in patients who are not suitable for EVL treatment, EIS or EVL can be repeated at an interval of 2 to 4 weeks after the first EIS until the varicose veins disappear or basically disappear. The commonly used hardener is lauryl alcohol (10 ml vs. 100 mg).

#### Endoscopic tissue adhesive injection

Mainly used for patients with GOV2 hemorrhage. The commonly used tissue adhesive is n-butyl cyocyanacrylate, and the sandwich Sandwich method is used for intravenous injection. Generally, one injection can completely block varicose veins.

### DT group

The main drugs used for treatment are portal pressure lowering drugs, antibacterial drugs, proton pump inhibitors (PPIs) and other symptomatic treatment drugs.

#### Portal pressure lowering drug

Teripressin, the dose selected 2 ~ 12 mg/d; Somatostatin, 250 ~ 500 μg/h; Octreotide, dosage selected 25 ~ 50 μg/h. The above drugs were administered by continuous intravenous infusion, with a general course of 3 to 5 days.

#### Antimicrobials

All patients were given a broad spectrum of antimicrobials intravenously, and if there was evidence of infection, the grade of antimicrobials would be increased according to the corresponding laboratory results, and/or the course of treatment would be extended appropriately.

PPIs: Generally choose omeprazole, Rabeprazole and other drugs, usually 40 to 80 mg/d intravenous injection or 8 mg/h continuous intravenous infusion, the general course of 5 to 7 days.

#### Other drugs

According to the clinical manifestations of patients, hemostatic drugs such as intravenous hemocoagulase and vitamin K1 are selected. Apply for transfusion of blood and/or blood products if the patient has indications for transfusion. After active bleeding was controlled for 24 h, lactulose was administered orally or by enema, and anemia correction drugs were given if necessary.

### Evaluation of curative effect and rebleeding

#### Evaluation of treatment failure^6^

One of the following manifestations: (i) Vomiting of fresh blood or more than 100 ml of fresh blood drawn by nasogastric tube ≥ 2 h after endoscopic therapy or drug therapy; (ii) Hemorrhagic shock occurs; (iii) Without transfusion, hemoglobin decreased by 30 g/L (hematocrit decreased by about 9%) during any 24 h period.

#### Signs of rebleeding^6^

Clinically significant active bleeding events after bleeding control (hematemesis, black stool, or blood in the stool; Systolic blood pressure decreased > 20 mmHg or heart rate increased > 20 beats/min; Hemoglobin decreased > 30 g/L in the absence of transfusion). (i) Early rebleeding: EVB appears within 120 h to 6 weeks after blood control; (ii) Delayed rebleeding: EVB occurs after 6 weeks of bleeding control; Non-EVB patients were not included.

### Statistical analysis

The 1:1 PSM analysis was performed using SPSS25.0 software (SPSS Inc.), and the caliper value was set to 0.02. Matching factors included sex, age, smoking history, drinking history, underlying disease, and Child–Pugh rating. Continuous variables were expressed as mean ± SD ($\overline{x }$ ± *s*), and two independent sample T-tests were used for comparison between groups. The categorical variables were expressed as frequency and percentage (%), and the Chi-square test or Fisher test was used for comparison between groups. *P* < 0.05 was considered statistically significant.

## Results

### Propensity score matching

There were significant differences in smoking and drinking history before PSM between the two groups. PSM included gender, age, smoking history, drinking history, underlying diseases, Child–Pugh grade and other factors, and 286 cases in the ET group and 286 cases in the DT group were successfully matched 1:1, with no statistically significant differences in confounding variables (see Table 1).

**Table 1:** Comparison of baseline information before and after propensity score matching between the two groups (cases (%)/$\overline{x }$ ± *s*).

Characteristics	Before PSM			After PSM		
	ET group (n = 586)	DT group (n = 379)	*P*	ET group (n = 286)	DT group (n = 286)	*P*
Sex			0.665			0.542
Male	377 (64.33)	249 (65.70)		187 (65.38)	180 (62.94)	
Female	209 (35.67)	130 (34.30)		99 (34.62)	106 (37.06)	
Age (years)	53.18 ± 10.81	63.08 ± 11.01	0.511	59.76 ± 9.95	59.85 ± 10.46	0.551
Smoking history			0.000			0.600
Yes	147 (25.09)	164 (43.27)		105 (36.71)	99 (34.62)	
No	439 (74.91)	215 (56.73)		181 (63.29)	187 (65.38)	
Drinking history			0.000			0.393
Yes	160 (27.30)	174 (45.91)		119 (41.61)	109 (38.11)	
No	426 (72.70)	205 (54.09)		167 (58.39)	177 (61.89)	
Basic diseases			0.145			0.452
Yes	278 (47.44)	198 (52.24)		149 (52.10)	140 (48.95)	
No	308 (52.56)	181 (47.76)		137 (47.90)	146 (51.05)	
Child–Pugh grade			0.057			0.064
A	31 (5.29)	9 (2.37)		7 (2.45)	9 (3.15)	
B	447 (76.28)	289 (76.25)		205 (71.68)	226 (79.02)	
C	108 (18.43)	81 (21.37)		74 (25.87)	51 (17.83)	

### Prognosis comparison

The clinical data of the two groups of patients after PSM are shown in Table 2. The short-term treatment success rate of ET was significantly better than that of DT (94.77% vs. 47.39, *P*< 0.001). Compared with the DT group, the rebleeding rate in ET was 26.83% vs. 44.60%, *P* < 0.001; Early bleeding rate: 1.4% vs. 14.69%, *P* < 0.001; The rate of delayed rebleeding was 21.68% vs. 31.82%, *P* = 0.006) and the rate of death within 3 months after treatment was lower (3.15% vs. 11.89%, *P*< 0.001). There was no significant difference in the mean length of stay between the two groups (11.45 ± 2.29 vs. 11.48 ± 2.30, *P* = 0.756), as shown in Table 2.

**Table 2:** Clinical prognostic data (cases (%)/$\overline{x }$ ± *s*).

Characteristics	ET group (n = 286)	DT group (n = 286)	*P*
Outcome			0.000
Success	274 (94.77)	176 (47.39)	
Failure	12 (5.23)	110 (52.61)	
Length of stay(days)	11.45 ± 2.29	11.48 ± 2.30	0.756
Rebleeding			0.000
Yes	65 (26.83)	121 (44.60)	
No	221 (73.17)	165 (55.40)	
Early rebleeding			0.000
Yes	4 (1.40)	42 (14.69)	
No	282 (98.60)	244 (85.31)	
Delayed rebleeding			0.006
Yes	62 (21.68)	91 (31.82)	
No	224 (78.32)	195 (68.18)	
Death			0.000
Yes	9 (3.15)	34 (11.89)	
No	277 (96.85)	252 (88.11)	

## Discussion

At present, the treatment principles for EVB are mainly to correct hypovolemic shock while effectively controlling bleeding and preventing bleeding related complications^7^. Some studies have reported that TIPS ranked first in the prevention of EVB secondary prevention of rebleeding, and carvedilol has shown outstanding efficacy in improving survival, but EVL + NSBB does not seem to be ideal for preventing rebleeding and extending survival in patients with EVB^8^. As a common method for the treatment of esophageal and gastric variceal hemorrhage recommended by several relevant guidelines and consensus, both endoscopic therapy and drug therapy have certain curative effects^9–13^. However, for the comparison of the efficacy of endoscopic therapy or drug therapy alone, the current reports have not reached a consistent conclusion. The short-term treatment effect evaluation, whether rebleeding will occur after treatment and the probability of death after treatment can reflect the efficacy to some extent.

In this study, we used PSM to compare the effects of endoscopic therapy and drug therapy on prognosis and rebleeding in patients with EVB. We found that the success rate of short-term hemostasis in the ET group was significantly better than that in the DT group, indicating that ET have certain advantages in short-term efficacy, which can reduce the occurrence of hemorrhagic shock. Our present study shows that ET can effectively reduce the rate of rebleeding and mortality within 3 months after treatment, suggesting that ET are superior in improving overall prognosis and delaying patient survival. However, there was no significant difference in length of stay between the two groups (*P* = 0.756).

Previous research reports have confirmed the effectiveness of EVL in the treatment of esophageal varices, but there does not seem to be much reporting on the comparison of the efficacy of EVB with the three different endoscopic treatments mentioned in our research^14^. Therefore, we further analyzed the influence of different endoscopic treatment methods on the clinical prognosis of EVB patients. The results showed that EVL seemed to be superior to the other two methods in reducing rebleeding in patients with EVB, but there was no significant difference between them in short-term hemostatic effect, length of hospital stay, and whether death occurred (see Supplementary Table 1), which was similar to the results of previous studies^15,16^.

There are some limitations and shortcomings in this study: First, due to the single-center data source of the included study, the sample size and clinical data of the final included study are relatively limited for retrospective study, and the results may be biased; Second, although endoscopic treatment of EVB has short-term efficacy advantages and can effectively reduce the incidence of rebleeding and mortality within 3 months, due to the short follow-up time of the two groups of patients, further studies are needed to determine whether this difference will affect patients' long-term survival or other clinical indicators (As our long-term follow-up is still ongoing, data are not yet available).

## Conclusions

In summary, successful hemostasis and reducing the occurrence of rebleeding are the key to effective treatment of EVB. According to our data, ET have a significant advantage over DT in successful hemostasis in the short term, and the probability of rebleeding events (whether early or delayed rebleeding) in ET seems to be much lower than that of DT. In addition, compared with the DT group, patients in the ET group had a lower mortality rate within 3 months after treatment. Therefore, for the patients with EVB, endoscopic treatment should be given in the case of excluding related contraindications to reduce the occurrence of rebleeding and improve the prognosis. However, more clinical studies with more data are needed to confirm which endoscopic therapy is more effective in alleviating or curing EVB.

### Supplementary Information

Supplementary Figure 1.

Supplementary Table 1.

Supplementary Table 2.

## Data Availability

The datasets generated and/or analysed during the current study are not publicly available due the raw data of this study contains the identity information of the subjects, which may involve their privacy, and the case browsing system of the hospital is not open to the public, but are available from the corresponding author on reasonable request.
