# Supplementary material for: Effect of endoscopic therapy and drug therapy on prognosis and rebleeding in patients with esophagogastric variceal bleeding
Source: Sci Rep. 2024 Mar 28;14:7364. doi: 10.1038/s41598-024-57791-8 (PMC10978843; doi:10.1038/s41598-024-57791-8)
Supplement: Supplementary file 1 — Supplementary Figure 1. [file 41598_2024_57791_MOESM1_ESM.docx]

**Supplementary Figure 1.** Flowchart of research methods.
