# Supplementary material for: Effect of endoscopic therapy and drug therapy on prognosis and rebleeding in patients with esophagogastric variceal bleeding
Source: Sci Rep. 2024 Mar 28;14:7364. doi: 10.1038/s41598-024-57791-8 (PMC10978843; doi:10.1038/s41598-024-57791-8)
Supplement: Supplementary file 2 — Supplementary Table 1. [file 41598_2024_57791_MOESM2_ESM.docx]

**Supplementary Table 1** Effect of three endoscopic therapy methods on clinical prognosis (cases (%)/$\bar{x}$ ± *s*).

| **Characteristics** | **Methods of endoscopic treatment** | | | |
| --- | --- | --- | --- | --- |
|  | **EVL（n=192）** | **EIS（n=56）** | **ETAI（n=38）** | **P** |
| Outcome |  |  |  | 0.075 |
| Success | 187(97.40) | 53(94.64) | 34(89.47) |  |
| failure | 5(2.60) | 3(5.36) | 4(10.53) |  |
| Length of stay(days) | 11.23±2.13 | 11.79±2.68 | 12.05±2.31 | 0.080 |
| Rebleeding |  |  |  | 0.000 |
| Yes | 23(11.98) | 21(37.50) | 17(44.74) |  |
| No | 169(88.02) | 35(62.50) | 21(55.26) |  |
| Early rebleeding |  |  |  | 0.072 |
| Yes | 1(0.52) | 1(1.79) | 2(5.26) |  |
| No | 191(99.48) | 55(98.21) | 36(94.74) |  |
| Delayed rebleeding |  |  |  | 0.046 |
| Yes | 34(17.71) | 15(26.79) | 13(34.21) |  |
| No | 158(82.29) | 41(73.21) | 25(65.79) |  |
| Death |  |  |  | 0.338 |
| Yes | 4(2.08) | 3(5.36) | 2(5.26) |  |
| No | 188(97.92) | 53(94.64) | 36(94.74) |  |
