# Supplementary material for: Effect of endoscopic therapy and drug therapy on prognosis and rebleeding in patients with esophagogastric variceal bleeding
Source: Sci Rep. 2024 Mar 28;14:7364. doi: 10.1038/s41598-024-57791-8 (PMC10978843; doi:10.1038/s41598-024-57791-8)
Supplement: Supplementary file 3 — Supplementary Table 2. [file 41598_2024_57791_MOESM3_ESM.docx]

**Supplementary Table 2** Cause of death.

| Cause of death | Number of death |
| --- | --- |
| Multiple organ failure | 16 |
| Hemorrhagic shock | 21 |
| septic shock | 5 |
| Hepatic encephalopathy | 1 |
